# A Meta-Analysis of Risk Factors for Post-Traumatic Stress Disorder (PTSD) in Adults and Children after Earthquakes

**DOI:** 10.3390/ijerph14121537

**Published:** 2017-12-08

**Authors:** Bihan Tang, Qiangyu Deng, Deborah Glik, Junqiang Dong, Lulu Zhang

**Affiliations:** 1 Department of Health Service, College of Health Service, Second Military Medical University, Shanghai 200433, China; mangotangbihan@126.com (B.T.); smmudqy@163.com (Q.D.); DJQdxyz@163.com (J.D.); 2 Department of Community Health Sciences, Fielding School of Public Health, University of California, Los Angeles, CA 90095, USA; dglik@ucla.edu

**Keywords:** PTSD, risk factors, children, adults, earthquakes

## Abstract

PTSD is considered the most common negative psychological reactions among survivors following an earthquake. The present study sought to find out the determinants of PTSD in earthquake survivors using a systematic meta-analysis. Four electronic databases (PubMed, Embase, Web of Science, and PsycInfo) were used to search for observational studies about PTSD following earthquakes. The literature search, study selection, and data extraction were conducted independently by two authors. 52 articles were included in the study. Summary estimates, subgroup analysis, and publication bias tests were performed on the data. The prevalence of PTSD after earthquakes ranged from 4.10% to 67.07% in adults and from 2.50% to 60.00% in children. For adults, the significant predictors were being female, low education level or socio-economic status, prior trauma; being trapped, experiencing fear, injury, or bereavement during the disaster. For children, the significant predictors were being older age, high education level; being trapped, experiencing fear, injury, or bereavement, witnessing injury/death during the earthquakes. Our study provides implications for the understanding of risk factors for PTSD among earthquake survivors. Post-disaster mental health recovery programs that include early identification, on-going monitoring, and sustained psychosocial support are needed for earthquake survivors.

## 1. Introduction

Earthquakes have the greatest destructive effect among all natural disasters [1], as they cause not only physical impairments, but also psychological stresses among victims. Over the past several decades, earthquakes have drawn attention for their frequent occurrence and massive destruction [2]. 

Post-traumatic stress disorder (PTSD) refers to a delayed but lasting psychological stress disorder caused by exposure to trauma, and it is considered the most common negative psychological reactions among survivors following an earthquake [3]. To have PTSD according to the Diagnostic and Statistical Manual of Mental Disorders 5th Edition (DSM-V), a person must first experience a life threatening traumatic event, an event outside the realm of daily human existence that evokes fear, helplessness, and/or horror [4]. These include a persistent re-experiencing of the event, a persistent avoidance of stimuli associated with the event, and the general numbing of responses to stimuli, as well as persistent symptoms that indicate emotional arousal or stress response [2,5]. Symptoms must last at least for 1 month and cause significant impairment in functioning [6].

PTSD was first included in the third edition of the Diagnostic and Statistical Manual of Mental Disorders (DSM-III) by American Psychiatric Association in 1980, and it continued to be listed in subsequent editions, that is, DSM-III-Revised (DSM-III-R), DSM-IV, DSM-IV-text revision (DSM-IV-TR), and DSM-V [4]. Posttraumatic stress disorder differs from other anxiety disorders in that the former is caused by the experience of a traumatic event [7]. It is also necessary for us to distinguish acute stress disorder (ASD) from PTSD. The primary difference between ASD and PTSD is the former’s emphasis on dissociative reactions to the trauma [8]. The requisite symptoms of ASD include re-experiencing the event, marked avoidance, marked anxiety or increased arousal, and evidence of significant distress or impairment. However, ASD only lasts for 2 days to 4 weeks, after which, if symptoms persist, a diagnosis of PTSD should be considered [8].

There are great variations in rates of PTSD currently reported in the wake of earthquakes. For example, 6 and 12 months after the Wenchuan earthquake in China, the prevalence rates were 11.2% and 13.4%, respectively, among the children in the town of Qushan, in Beichuan County [9]. Another study that focused on the adult population aged over 18 years was conducted 30 months after Pakistan’s 2005 earthquake, and it estimated the prevalence of PTSD to be as high as 41.3% [2]. Furthermore, the prevalence of PTSD was 24.6% among the survivors in the Nazon area of Port-au-Prince, 2 to 3.5 months after the 2010 Haiti earthquake [10]. Besides the different prevalance of PTSD among earthquake survivors, their predictors were also not consistent. For example, some studies found that being old was a risk factor for PTSD among adult earthquake survivors while some studies made the opposite conclusions [11,12,13,14]. The same phenomenon existed in other factors, such as marital status, injured as a result of earthquake and so on [2,15,16,17]. Variations in the prevalence and predictors of PTSD have been noted and attributed to differences in study measurements and time of assessment, as well as differences in age, gender, disease history, and cultural background of the samples, and so on [18]. As there is no single dominant determinant that predicts PTSD after an earthquake, it is important to study the risk factors involved. 

The onset of PTSD has been studied for more than 20 years and several meta-analyses have been carried out on risk factors for PTSD in different populations. For example, Ozer et al. [19] conducted a meta-analysis on seven separate predictors from 68 studies for PTSD among adults. Trickey et al. estimated the population effect sizes of 25 potential risk factors for PTSD in children and adolescents aged 6–18 years across 64 studies (*n* = 32,238) [20]. These have focused on different kinds of populations such as trauma-exposed adults [21], rescue workers [22], HIV-positive women [23], refugees and internally displaced persons [24], military personnel, and veterans [25]. To date, however, there has been no meta-analysis of risk factors for PTSD in populations specifically affected by earthquakes. 

While not all people who have suffered a qualifying trauma go on to develop a disorder, investigators have documented basic characteristics, trauma-related, and post-trauma risk factors that may increase vulnerability to a PTSD [26]. Similarly, risk factors for PTSD among earthquake survivors can also be divided into these three categories but quite different in detail. Earthquake survivors have their specific factors which resulted in PTSD symptoms, such as earthquake-related experience (i.e., trapped in an earthquake, injured by the earthquake) and post-earthquake characteristics (i.e., house damage and property loss as a result of earthquakes). Although there are many separate articles focusing on predictors of PTSD among earthquake survivors, their results are not always consistent. It is essential to make a meta-analysis to elaborate on the determinants of PTSD on earthquake survivors.

Considering the need to better understand survivors in face of an earthquake, and to assist them more effectively, this study aimed to identify the determinants of PTSD in earthquake survivors using a systematic meta-analysis of observational studies. In all, our study can add essential information to the better preparedness and implementation of tailored treatments in earthquake-stricken areas to reduce symptoms and aid post-disaster recovery. 

## 2. Methods

### 2.1. Search Strategy

Observational studies (case-control, cross-sectional, and cohort studies) on risk factors for PTSD among earthquake survivors published in English between 1980 (the year PTSD was first included in the Diagnostic and Statistical Manual of Mental Disorders, DSM) and April 2016 were included in this meta-analysis. The following four psychological and medical literature databases were searched: PubMed, Embase, PsycInfo, and Web of Science. Search terms entered into the literature databases included combinations of the following: PTSD or post-traumatic stress disorder; earthquake(s); and risk, predictor, or prediction. A predictor or risk factor was operationally defined as any variable examined as a potential contributor to variability in PTSD symptom levels or diagnostic status. In addition, a manual search of references cited in all relevant original and review articles was conducted. For any full texts not available, we attempted to obtain information from the authors by email. This literature search yielded 886 published articles, which were then reviewed for inclusion in the meta-analysis using various inclusion and exclusion criteria. 

### 2.2. Inclusion and Exclusion Criteria

To be eligible for inclusion in the meta-analysis, studies had to meet the following criteria: (a) was an epidemiological investigation on risk factors for PTSD among earthquake survivors; (b) reported the odds ratios (ORs) or relative risks (RRs) and corresponding 95% confidence intervals (CIs) for risk factors in the development of PTSD in the study population; (c) included PTSD risk factors selected; and (d) defined PTSD with validated scales. The term “validated scale” referred to generalized PTSD scales that have been verified to exhibit good reliability and validity, such as DSM-IV, the civilian version of the PTSD Checklist (PCL), Impact of event scale-Revised (IES-R), Davidson Trauma Scale (DTS), and so on. Articles were excluded on any of the following grounds: (a) the study measured only the acute trauma response (e.g., Acute Stress Disorder or PTSD measured before one month post-trauma) rather than PTSD, which, according to the DSM-IV-TR, can be diagnosed only after one month [4]; (b) the study used only a categorical measure of PTSD—in other words, they included individuals meeting full diagnostic criteria and those with less severe post-traumatic symptoms or partial PTSD (e.g., “subsyndromal PTSD”) in the same comparison group, and contrasted them with a group exposed to the same event, but without PTSD; (c) the study population consisted entirely of individuals already suffering from PTSD or from a specific comorbid psychiatric disorder (e.g., depression, attention deficit hyperactivity disorder, substance abuse, learning disabilities) or having committed a violent offense, which would limit the generalizability of the results; (d) the study did not specifically assess DSM-defined PTSD symptoms (e.g., studies that reported only general symptoms); (e) the study contained insufficient data to calculate univariate effect sizes, and such data could not be obtained from the study author; (f) the article was a review, did not present new data, or only presented qualitative analyses; (g) the primary aim of the study was to investigate treatment efficacy; and (h) the study used a single-case design. Studies in which most of the study sample was less than 18 years old were classified as child studies; otherwise, they were classified as adult studies. Finally, if more than one article reported data from the same sample, then the most recent and complete article was included in our meta-analysis. All eligible studies were carefully reviewed by two authors (Bihan Tang and Qiangyu Deng) to ensure decision-rule consistency, with 100% agreement. 

### 2.3. Data Extraction and Quality Assessment

Data extraction was performed by two investigators (Bihan Tang and Qiangyu Deng) independently. The following information was extracted from each eligible study: first author’s surname, year of publication, earthquake location, earthquake year, magnitude, population type, study design, PTSD diagnosis, sample size, PTSD prevalence, research time after the earthquake, gender of the participants, estimated effect size (OR/RR), corresponding 95% CI, and covariates adjusted for in the statistical analysis. If a study reported several multivariable-adjusted effect estimates, we selected the estimate that adjusted for the most potential confounding variables, when considering the major risk factors for PTSD among earthquake survivors. Quality assessments of each study identified were conducted independently by two investigators (Bihan Tang and Qiangyu Deng) using an 11-item instrument recommended by the Agency for Healthcare Research and Quality (AHRQ) for cross-sectional studies [27] and the 9-star Newcastle-Ottawa Scale (NOS) for cohort and case-control studies [28]. Studies with eight stars or more on the AHRQ and NOS were considered to be of high quality. Where the two raters’ quality assessments differed, the original articles were re-examined along with two more co-authors, until a final quality rating was agreed upon. 

### 2.4. Classification of Risk Factors

According to previously published studies, risk factors for PTSD among children and adults after earthquakes were divided into three categories: basic characteristics (including age, gender, education, marital status, religious beliefs, ethnicity, prior trauma, socio-economic status and disease history), trauma characteristics (including being trapped; experiencing fear, injury, or bereavement, e.g., losing close friends or family members; or witnessing injury/death as a result of the earthquakes), and post-trauma characteristics (including amount of social support, employment, loss of property, house damage and involved in rescue) [29].

### 2.5. Statistical Analysis

We examined risk factors for PTSD in earthquake survivors based on the ORs and 95% CIs reported in each study. A random-effects model [30] which assumes that the real potential effect varies among included studies, was used to estimate the pooled RRs with 95% CIs. Heterogeneity between studies was evaluated using the *χ*^2^ test and *I*^2^ statistic, with *p* values lower than 0.05 indicating heterogeneity and higher *I*^2^ values indicating greater variability among trials than that which would be expected by chance alone (range: 0–100%) [31]. The thresholds of 25% and 50% in *I*^2^ test indicated low, moderate, and high heterogeneity, respectively. The probability of publication bias was assessed using Egger’s regression test, and values of *p* < 0.05 were considered statistically publication bias [32]. If publication bias was present, we tried to evaluate the effect of the publication bias using the trim and fill method. We separated out data collected on children from adults as research suggests the presence of somewhat different factors [29]. To examine the possible effects of these variables on the study results, we conducted analyses for the following subgroups analyses: high-quality studies (with a score of 8 or more), survivors from high magnitude earthquakes (magnitude of above 7). Having a long period between the research and the earthquake raises the possibility that participants may have been exposed to subsequent traumatic events in addition to the earthquake, which may confound the interpretation of the results. Therefore, we also excluded studies that were implemented a long time after the onset of the disaster (>6 months). When risk factors were for multi-categorical variables (e.g., age, educational level, fear, house damage, loss of property, and social support), we used the ORs of the highest versus the lowest category. If risk factors were continuous variables (such as age in some studies), they were excluded to avoid inaccuracy because it is not appropriate to combine the ORs from continuous and segmental data. Stata Version 12.0 software (Stata Corp., College Station, TX, USA) was used for all analyses and all statistical tests were two-tailed. Values of *p* < 0.05 were considered statistically significant.

## 3. Results

### 3.1. Characteristics of Studies

**Figure 1:**
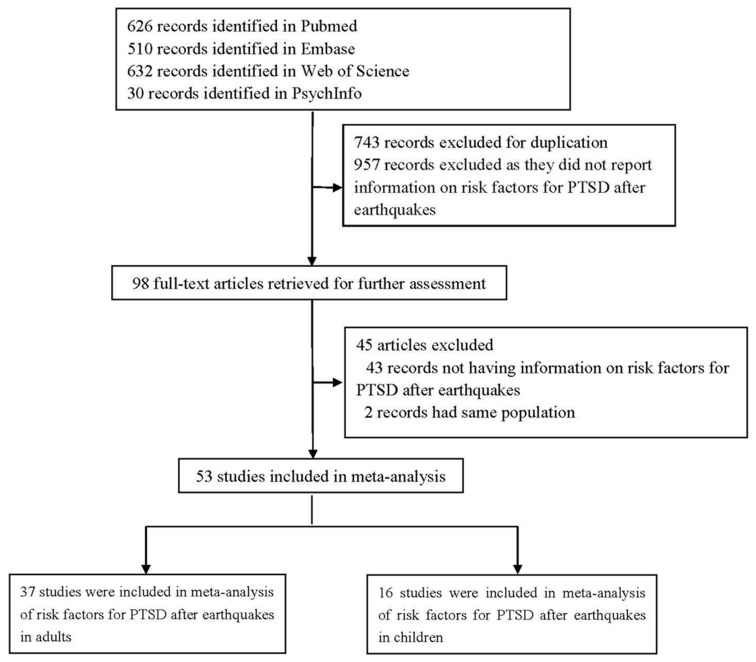
Search results and excluded/included studies.

Figure 1 shows the complete selection process. A total of 1798 records indexed until April 2016 were retrieved using our search strategy, and 744 records were discarded owing to duplication. We excluded another 957 articles after reading the titles and abstracts, and retained the remaining 97 articles for further evaluation by reading the full texts. Finally, we selected 52 full-text articles on risk factors for earthquake-related PTSD among survivors for our meta-analysis (Figure 1) [2,3,6,10,11,12,13,14,15,16,26,33,34,35,36,37,38,39,40,41,42,43,44,45,46,47,48,49,50,51,52,53,54,55,56,57,58,59,60,61,62,63,64,65,66,67,68,69,70,71,72]. Furthermore, 37 studies investigated the association between risk factors and PTSD in adult survivors of earthquakes, totaling 11,963 PTSD cases out of 56,722 participants. 15 studies investigated the association between risk factors and PTSD in child survivors of earthquakes, totaling 3461 PTSD cases out of 22,931 participants. Table A1 shows the general characteristics of the 52 studies included in the analysis. Sample sizes from individual studies ranged from 105 to 14,207.

### 3.2. Risk Factors for PTSD in Adults

**Figure 2:**
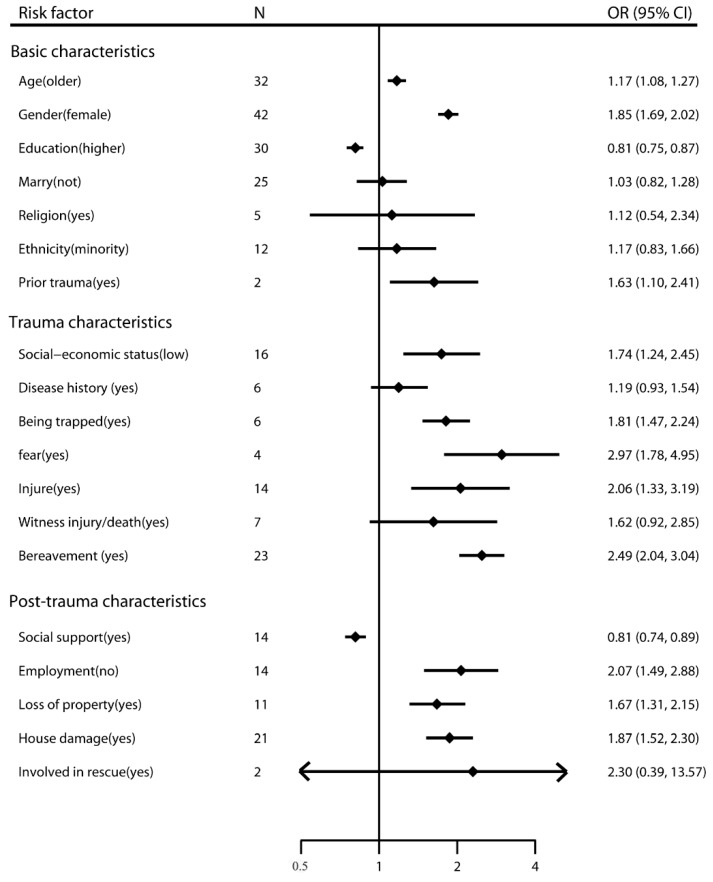
Risk factors for PTSD after earthquake in adults.

The prevalence of PTSD in adults after earthquakes ranged from 4.10% to 67.07%. The risk factors for earthquake-related PTSD among adult survivors have been presented in Table A2 and Figure 2. Regarding the basic characteristics of survivors, we found that being older, being female, having a high-level education, low socio-economic status, and prior trauma were significantly associated with PTSD after earthquakes, with pooled ORs of 1.17 (95% CI, 1.08–1.27), 1.85 (95% CI, 1.69–2.02), 0.81 (95% CI, 0.75–0.87), 1.74 (95% CI, 1.24–2.45) and 1.63 (95% CI, 1.10–2.41), respectively. However, heterogeneity was found for age (*I*^2^ = 73.2%, *p* < 0.001), gender (*I*^2^ = 51.1%, *p* < 0.001), education (*I*^2^ = 33.3%, *p* = 0.041), marriage (*I*^2^ = 79.6%, *p* < 0.001), religion (*I*^2^ = 72.0%, *p* = 0.006), ethnicity (*I*^2^ = 82.9%, *p* < 0.001), socio-economic status (*I*^2^ = 89.0%, *p* < 0.001), and prior trauma (*I*^2^ = 55.3%, *p* = 0.135). In addition, we found a publication bias for age (Egger’s test *p* = 0.013). Thus, after adjusting for publication bias, the OR for age was 1.05 (95% CI, 0.96–1.15), which was no longer a significant risk factor. The subgroup analyses showed inconsistencies in the results for age, socio-economic status and ethnicity, which should be interpreted with caution because of potential bias.

With regard to the trauma characteristics of survivors, people who experienced being trapped, or those who experienced fear, injury, or bereavement during a natural disaster were more likely to suffer from PTSD, with pooled ORs of 1.81 (95% CI, 1.47–2.24), 2.97 (95% CI, 1.78–4.95), 2.06 (95% CI, 1.33–3.19), and 2.49 (95% CI, 2.04–3.04), respectively. Heterogeneity was found for the characteristics of experiencing fear (*I*^2^ = 92.9%, *p* < 0.001), injury (*I*^2^ = 90.0%, *p* < 0.001), witnessing injury/death (*I*^2^ = 90.5%, *p* = 0.041), and bereavement (*I*^2^ = 70.8%, *p* < 0.001). The results were consistent according to the subgroup analyses, except for injury.

Finally, analysis of the post-trauma characteristics of survivors showed that social support (OR 0.81, 95% CI, 0.74–0.89), employment (OR 2.07, 95% CI, 1.49–2.88), loss of property (OR 1.67, 95% CI, 1.31–2.15), and house damage (OR 1.87, 95% CI, 1.52–2.30) were related to PTSD. However, heterogeneity was observed with reference to social support (*I*^2^ = 94.7%, *p* < 0.001), employment (*I*^2^ = 85.5%, *p* < 0.001), loss of property (*I*^2^ = 60.2%, *p* = 0.005), house damage (*I*^2^ = 60.6%, *p* < 0.001), and involvement in rescue (*I*^2^ = 98.1%, *p* < 0.001), suggesting that the findings on these variables showed inconsistencies. The subgroup analyses showed inconsistencies in the results for social support and involvement in rescue, which should be interpreted with caution because of potential bias. We also found a publication bias for loss of property (Egger’s test *p* = 0.005). Thus, after adjusting for publication bias, the OR was 1.30 (95% CI, 1.01–1.68).

### 3.3. Risk Factors for PTSD in Children

**Figure 3:**
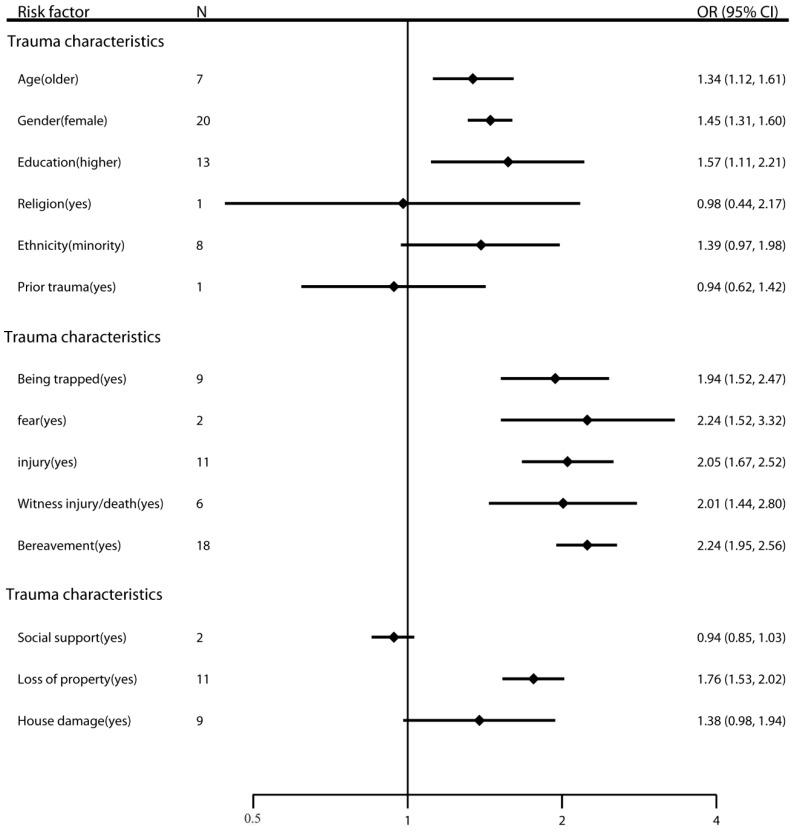
Risk factors for PTSD after earthquake in children.

The prevalence of PTSD in children after earthquakes ranged from 2.50% to 60.00%. The risk factors for PTSD after earthquakes in children have been presented in Table A3 and Figure 3. Regarding the basic characteristics of survivors, the pooled analysis showed that age (OR 1.34, 95% CI, 1.12–1.61), gender (OR 1.45, 95% CI, 1.31–1.60), and educational level (OR 1.57, 95% CI, 1.11–2.21) were associated with risk of PTSD. However, heterogeneity was found for educational level (*I*^2^ = 77.7%, *p* < 0.001). Furthermore, we found inconsistencies in educational level in the subgroup analyses.

The initial analysis of the trauma characteristics of survivors (i.e., before excluding low-quality and unadjusted studies) revealed that all five factors were associated with risk of PTSD; the pooled ORs were 1.94 (95% CI, 1.52–2.47) for being trapped, 2.24 (95% CI, 1.52–3.32) for experiencing fear, 2.05 (95% CI, 1.67–2.52) for experiencing injury, 2.01 (95% CI, 1.44–2.80) for witnessing injury/death, and 2.24 (95% CI, 1.95–2.56) for bereavement. However, heterogeneity was found for witnessing injury/death (*I*^2^ = 64.9%, *p* = 0.014), which also had a publication bias. After adjusting for publication bias, the OR for witnessing injury/death was 1.54 (95% CI, 1.12–2.13). Besides, the subgroup analyses showed inconsistencies in the results for experiencing fear, which should be interpreted with caution because of potential bias.

Finally, with regard to the post-trauma characteristics of survivors, only one factor (i.e., loss of property) was found to be a risk factor for the onset of PTSD (OR 1.76, 95% CI, 1.53–2.02). Heterogeneity was found for house damage (*I*^2^ = 55.1%, *p* = 0.023).

## 4. Discussion

To the best of our knowledge, this is the first meta-analysis focusing on the risk factors for PTSD in populations specifically affected by earthquakes. Our synthesis of the relevant published English-language articles provided strong evidence for risk factors of PTSD among earthquake survivors. This study analyzed 52 published observational studies (1 case-control, 1 cohort, and 50 cross-sectional studies, including a total of 79,653 people). A total of 19 risk factors of PTSD in the survivors of earthquakes were explored and categorized into the following three types: basic characteristics, trauma characteristics, and post-trauma characteristics. Fourteen (73.68%) risk factors were investigated in ten or more studies. Although an increasing number of researchers have been studying earthquake-related PTSD in the past decades, only a limited number of variables have been routinely investigated.

With regard to basic characteristics, one important risk factor for the development of PTSD in both children and adults was gender. This is consistent with a report of the WHO that also showed gender differences in the ability to cope with stress after disasters [73]. Genetic and biological factors are expected to play some role in these differences. For example, women are thought to be more sensitive to stress hormones, more sensitive to threats, less likely to use effective coping strategies, and more likely to interpret disasters more negatively than men are [17]. In addition, the traditional role of females in the society exposes them to greater stress, which may also render them less capable of changing their stressful environment. Gender was a common risk factor both in children and in adults. It is possible that the gender role socialization that begins at an early age may play a role as boys learn to suppressor deny psychological symptoms, whereas girls learn to reflect their feelings more and become more emotionally expressive [74]. Our results also suggested that older child survivors after earthquakes were more likely to develop PTSD than younger ones were. This may reflect the universal characteristics of child PTSD distribution after earthquakes, and further research is needed to investigate the psychosocial and biological mechanisms of older female children who are at high risk for PTSD after earthquakes. Adults with a low educational level and socio-economic status were at high risk of PTSD after earthquakes. For adults, level of education indirectly influences economic resources, social status, social networks, and health behavior [60]. Thus, those with a higher level of education and socio-economic status might use better coping methods because of their greater social and economic resources, and ultimately would be less impacted by earthquakes, which in turn reduces the prevalence of PTSD. However, as for children, high educational level indicated a higher risk of PTSD. Senior students might experience greater academic stress due to their heavier study load than junior students would, which could result in higher PTSD prevalence. Prior exposure to trauma was another risk factor of PTSD among adult survivors, which can be partly explained by the idea that the accumulation of violent traumatic events throughout the life course could increase the risk of mental disorders [10]. As for children, prior exposure to trauma was not a significant risk factor, since children may be too young to accumulate enough violent traumatic events to affect their psychological health status in an obvious sense. 

In terms of trauma characteristics, children and adults shared the following four risk factors: experience of being trapped, fear, injury, and bereavement. Being trapped is without a doubt an extremely traumatic experience. Therefore, the persistence of PTSD symptoms in this group seemed to be related to pervasive trauma reminders that were embedded in the circumstances after the earthquake. Reminders such as debris, destroyed buildings, and casualties served as ongoing reminders of the earthquake-related trauma and continually reactivated the symptoms [6]. Given that PTSD is a fear-based disorder, fear during the earthquake was a strong predictor of PTSD. Exposure-based treatments have proven to be highly effective in PTSD, and psychological interventions targeting fear in earthquake survivors are expected to lead to a reduction in traumatic stress symptoms [68]. The link between being injured and PTSD is possibly related to the severity of the injuries, which are often so severe after an earthquake that they result in amputation and disability [26]. The onset of disability is likely to reduce health-related quality of life in these people, and this loss of quality of life might lead to PTSD. Besides, people who were injured were always at high risk, reflecting both their high level of exposure to mortal danger and the added persistent reminder and additional stress accompanying an ongoing injury and the necessary rehabilitation associated with it [11]. In our study, most articles concluded that injury was a risk factor of PTSD and only two articles considered injury as a protective factor of PTSD. This phenomenon may be partly explained by study bias, and partly by the possibility that those who were injured often got more social support than those who were not injured, which made them less susceptible to PTSD. Bereavement removes part of the support resources from close relationships at a time of intense need, compounding the psychological stress and predisposing individuals to PTSD. This highlighted that the bereaved survivors should receive psychological screening and indicated interventions by priority. Witnessing injury or death was another risk factor of PTSD among child survivors. Children are more sensitive to traumatic images compared to adults. For adults, accumulation of life experience makes them more adaptable and calm when exposed to dramatic scenes [26]. However, the horrible memory, in which children witnessed people being crushed and wounded by collapsed and destroyed buildings, is often re-experienced several months or even several years later in their lives [62]. Furthermore, being direct witnesses of traumatic scenes might exert adverse effects on children’s cognitive and emotional functioning. 

With regard to post-trauma characteristics, loss of property was associated with a higher risk of PTSD both in children and in adults. With loss of property, children and their family members might face persistent financial problems and difficulties with living arrangements. Children tend to worry about the family’s situation and the future [75]. As for adults, the risk factors for the onset of PTSD also included house damage, unemployment, social support, and socio-economic status. It is reasonable to assume that the extent of dwelling destruction and unemployment due to an earthquake could reflect the strength of trauma to some degree. Moreover, unemployment and severe house damage resulted in people becoming unable to care for their families to the same extent that they could before the disaster, indicating that the implementation of social interventions such as income-generating activities and facilitating early return to work are critical. When facing forces of earthquakes, social relationships as well as material and spiritual encouragement from families and friends positively offset the negative effects of trauma. Social support could be helpful in PTSD intervention and prevention measures, and substantial social support should be encouraged to be offered by the government and other aid organizations.

It is important to note that our study had some limitations. This meta-analysis only included observational studies; most data were based on self-report measures, which can be prone to biases in sample selection, recall, and information evaluation, as well as confounding biases. There was also significant heterogeneity among the studies due to differences in sampling, design, measurement, and statistical analysis. This is not uncommon in reviews of observational studies. Furthermore, the initial search was restricted to publications in English language, and it is not known to what extent this limitation may have influenced the findings. Moreover, many of the variables included in the analyses were only examined in a small proportion of studies, which limits the conclusions that can be drawn about these risk factors. However, the present study does also help to highlight areas that warrant further investigations.

## 5. Conclusions

Despite the limitations outlined above, our study provides some implications for the theoretical understanding of PTSD among earthquake survivors. Firstly, as earthquakes occur increasingly frequently all over the world, these data help psychiatrists to identify the vulnerable population more effectively, which in turn would help them administer treatment in time. Secondly, our results suggest that PTSD in disaster survivors may persist over time; hence, there is a need to introduce feasible interventions at the community or primary mental health care level. The results may also provide clear intervention directions for government policy makers to establish programs to improve assessments, prevention efforts, and interventions for at-risk groups. In addition, these results can help clinicians understand the relevant risk factors and provide treatment prior to difficulties becoming chronic. Besides, general practitioners should be aware of PTSD symptoms, and careful consideration should be given to routine screening for PTSD during the reconstruction process following earthquakes. Above all, post-disaster mental health recovery programs that include early identification, on-going monitoring, prevention and intervention programs, and sustained psychosocial support are needed for the high-risk population of earthquake survivors.

## Appendix A

**Table A1:** General characteristic of the included studies with regard to risk factors for PTSD after earthquakes.

No.	Author	Publication Year	Country	Earthquake Year	Magnitude	Population	Study Design	Identification of PTSD	Sample Size	PTSD Prevalence %	Time after Disaster	Male %	Quality
1	Ali	2012	Pakistan	2005	7.6	adults	cross-section	Davidson Trauma Scale (DTS)	300	41.30	30 months	60.7	8
2	Altindag	2005	Turkey	1998	6.3	adults	cross-section	Diagnostic and Statistical Manual of Mental Disorders, Fourth Edition	105	42.00, 23.00	1, 13 months	47.6	8
3	Cairo	2010	Peru	2007	8.0	adults	cross-section	a Spanish translation of the civilian version of the PTSD Checklist (PCL)	298	25.20	5 months	31.88	7
4	Armenian	2000	Armenia	1988	6.9	adults	cohort	DSM-III-R	737	49.60	2 years	46.68	7
5	Cenat	2014	Haiti	2010	7.0	adults	cross-section	Impact of event scale—Revised (IES-R)	1355	36.75	30 months	51.29	8
6	Cenat	2015	Haiti	2010	7.0	children	cross-section	Impact of event scale—Revised (IES-R)	872	36.93	30 months	43.7	8
7	Cerda	2013	Haiti	2010	7.0	adults	cross-section	DSM-IV	1315	24.60	2–4 months	28.9	7
8	Chen	2007	China (Taiwan)	1999	7.3	adults	cross-section	The Davidson Trauma Scale (DTS)	6412	20.90	24 months	38.8	8
9	Chen	2012	China	2008	8.0	adults	cross-section	the CAPS for DSM-IV	287	22.65	36 months	35.9	7
10	Cheng	2014	China	2008	8.0	adults	case-control	the civilian version of the PTSD Checklist (PCL)	565	20.00	36 months	41.6	8
11	Cheng	2015	China	2008	8.0	adults	cross-section	Structured Clinical Interview for DSM-IV Axis I Disorders (SCID-I/NP)	182	39.60	12 months	34.8	8
12	Chou	2005	China (Taiwan)	1999	7.3	adults	cross-section	DSM-IV	442	7.90	6 months	48.4	8
13	Chou	2007	China (Taiwan)	1999	7.3	adults	cross-section	DSM-IV	216	8.30	6, 24, 36 months	45.8	8
14	Cofini	2015	Italy	2009	6.3	adults	cross-section	Davidson Trauma Scale (DTS)	281	43.00	12 months	46	9
15	Eksi	2007	Turkey	1999	7.4	children	cross-section	The Clinician-Administered Post-Traumatic Stress Disorder Scale (CAPS)	160	60.00	6–20 weeks	36.25	7
16	Fan	2015	China	2008	8.0	children	cross-section	The Posttraumatic Stress Disorder Self-Rating Scale (PTSD-SS)	1573	21.00, 23.35, 13.50, 14.70	6, 12, 18, 24 months	45.77	9
17	Fan	2011	China	2008	8.0	children	cross-section	The Posttraumatic Stress Disorder Self-Rating Scale (PTSD-SS)	2081	15.80	6 months	45.9	9
18	Gigantesco	2013	Italy	2009	6.3	adults	cross-section	Mini-International Neuropsychiatric Interview (MINI)	957	4.10	14–19 months	49.2	8
19	Guo	2015	China	2008	8.0	adults	cross-section	Impact of Event Scale-Revised (IES-R)	1362	22.10	6 months	36	7
20	Guo	2014	China	2008	8.0	adults	cross-section	Impact of Event Scale-Revised (IES-R)	1066	58.20, 22.10, 19.80, 8.00	2, 8, 14, 26, 44 months	37.3	7
21	Hashmi	2011	Pakistan	2005	7.6	adults	cross-section	the civilian version of the PTSD Checklist (PCL)	361	51.50	6 months	43	8
22	Hikichi	2016	Japan	2011	9.0	adults	cross-section	the Screening Questionnaire for Disaster-Related Mental Health	3567	11.40	30 months	43.5	8
23	Jia	2010	China	2008	8.0	children	cross-section	the self-report Child Posttraumatic Stress Disorder Reaction Index (CPTSD-RI)	596	12.40	15 months	49.8	9
24	Jia	2010	China	2008	8.0	adults	cross-section	the self-report Child Posttraumatic Stress Disorder Reaction Index (CPTSD-RI)	276	15.20	15 months	53.6	7
25	Jin	2015	China	2010	7.1	children	cross-section	the civilian version of the PTSD Checklist (PCL)	459	10.90, 5.80	6, 24 months	40.50	8
26	Jin	2014	China	2010	7.1	children	cross-section	the civilian version of the PTSD Checklist (PCL)	850	8.94	36 months	44.8	7
27	Kadak	2013	Turkey	2011	7.2	children	cross-section	Child Posttraumatic Stress Disorder–Reaction Index (CPTSD-R I)	738	40.69	6 months	55.01	8
28	Kun	2009	China	2008	8.0	adults	cross-section	The Harvard Trauma Questionnaire (HTQ)	446	45.50	2.5 months	49.5	10
29	Kun	2013	China	2008	8.0	adults	cross-section	the Harvard Trauma Questionnaire (HTQ)	992	47.30	3 months	49.2	10
30	Lau	2010	China	2008	8.0	children	cross-section	the Children’s Revised Impact of Event Scale	3324	22.30	1 month	54.3	8
31	Lee	2009	China (Taiwan)	1999	7.3	adults	cross-section	DSM-IV	196	38.80	5 months	54.6	7
32	Liu	2011	China	2008	8.0	children	cross-section	Trauma Symptom Checklist for Children-Alternate Version (TSCC-A)	330	11.20, 13.40	6, 12 months	50	9
33	Liu	2012	China	2008	8.0	adults	cross-section	the civilian version of the PTSD Checklist (PCL)	9556	4.50	6 months	45.3	6
34	Liu	2010	China	2008	8.0	children	cross-section	the civilian version of the PTSD Checklist (PCL)	1474	5.70	12 months	45	8
35	Ma	2011	China	2008	8.0	children	cross-section	DSM-IV	3208	2.50	6 months	47.9	7
36	Pan	2015	China	2008	8.0	children	cross-section	Chinese version of the Impact of Event Scale–Revised (IES-R)	362	29.60	36 months	43.6	8
37	Peng	2009	China	2008	8.0	adults	cross-section	the Harvard Trauma Questionnaire (HTQ)	446	45.50	3 months	50.9	8
38	Priebe	2009	Italy	2002	5.4	adults	cross-section	The Breslau scale	1680	14.50	6 months	44.88	7
39	Roncone	2013	Italy	2009	6.3	adults	cross-section	the Impact of Events Scale-revised (IES-R)	91	56.00	6 months	42.9	
40	Tian	2014	China	2008	8.0	children	cross-section	DSM-IV	4604	5.70	36 months	56.8	7
41	Tural	2004	Turkey	1998	6.3	adults	cross-section	DSM-IV	910	25.40	4–12 months	36.3	7
42	Wang	2011	China	2008	8.0	adults	cross-section	Posttraumatic Stress Disorder Self-rating Scale (PTSD-SS)	409	62.80	1 month	50.12	9
43	Wang	2012	China	2008	8.0	children	cross-section	Children’s Revised Impact of Event Scale (CRIES-13)	1841	28.40	10 months	48.7	9
44	Wen	2012	China	2008	8.0	adults	cross-section	the civilian version of the PTSD Checklist (PCL)	1206	4.46	36 months	46.93	9
45	Xu	2011	China	2008	8.0	adults	cross-section	the civilian version of the PTSD Checklist (PCL)	367	48.20	12 months	53.4	8
46	Xu	2011	China	2008	8.0	adults	cross-section	the civilian version of the PTSD Checklist (PCL)	2080	67.07	12–16 months	47.26	8
47	Zhang	2015	China	2008	8.0	adults	cross-section	the civilian version of the PTSD Checklist (PCL)	684	9.20	60 months	42.1	8
48	Zhang	2015	China	2008	8.0	adults	cross-section	the civilian version of the PTSD Checklist (PCL)	360	10.30	36 months	55.6	9
49	Zhang	2011	China	2008	8.0	adults	cross-section	the civilian version of the PTSD Checklist (PCL)	1181	26.30	12 months	37.3	10
50	Zhang	2012	China	2008	8.0	adults	cross-section	the civilian version of the PTSD Checklist (PCL)	274	26.30	14 months	38	8
51	Zhang	2012	China	2010	7.1	adults	cross-section	the civilian version of the PTSD Checklist (PCL)	505	33.70	3–4 months	53.5	8
52	Zhou	2013	China	2008	8.0	adults	cross-section	a Chinese version of the Structured Clinical Interview for Diagnostic and Statistical Manual (DSM)-IV axis I disorders (SCID-I/P)	14,207	15.57	6 months	48.42	8

* No.: Number.

**Table A2:** Risk Factors for PTSD after earthquakes in adults.

Factors	All Studies				High Quality		Adjustment		Within 6 Months		High Magnitude	
	*n*	OR (95% CI)	*I*^2^ (*p* Value)	Egger Test	*n*	OR (95% CI)	*n*	OR (95% CI)	*n*	OR (95% CI)	*n*	OR (95% CI)
Basic characteristics												
Age (older)	32	1.17 (1.08–1.27)	73.2% (*p* < 0.001)	*p* = 0.013 *	20	1.07 (0.98–1.17)	25	1.12 (1.03–1.22)	12	1.49 (1.13–1.97)	26	1.16 (1.07–1.27)
Gender (female)	42	1.85 (1.69–2.02)	51.1% (*p* < 0.001)	*p* = 0.855	28	1.94 (1.74–2.15)	33	1.85 (1.69–2.03)	16	2.01 (1.67–2.42)	34	1.82 (1.66–1.99)
Education (higher)	30	0.81 (0.75–0.87)	33.3% (*p* = 0.041)	*p* = 0.550	18	0.86 (0.78–0.95)	22	0.83 (0.76–0.90)	12	0.78 (0.67–0.89)	22	0.81 (0.74–0.88)
Marry (not)	25	1.03 (0.82–1.28)	79.6% (*p* < 0.001)	*p* = 0.226	14	1.35 (0.93–1.97)	19	1.04 (0.80–1.35)	11	0.95 (0.52–1.72)	14	1.35 (0.93–1.97)
Religion (yes)	5	1.12 (0.54–2.34)	72.0% (*p* = 0.006)	*p* = 0.661	5	1.12 (0.54–2.34)	3	0.97 (0.27–3.52)	1	2.54 (0.86–7.52)	4	1.07 (0.42–2.76)
Ethnicity (minority)	12	1.17 (0.83–1.66)	82.9% (*p* < 0.001)	*p* = 0.490	11	1.19 (0.83–1.73)	8	1.30 (0.83–2.05)	3	1.77 (1.12–2.79)	12	1.17 (0.83–1.66)
Prior trauma (yes)	2	1.63 (1.10–2.41)	55.3% (*p* = 0.135)	–	1	2.07 (1.33–3.23)	1	1.38 (1.03–1.85)	1	2.07 (1.33–3.23)	1	2.07 (1.33–3.23)
Socio-economic status (low)	16	1.74 (1.24–2.45)	89.0% (*p* < 0.001)	*p* = 0.532	9	2.91 (1.56–5.41)	15	1.68 (1.18–2.40)	3	1.73 (0.71–4.17)	16	1.74 (1.24–2.45)
Disease history (yes)	6	1.19 (0.93–1.54)	0.0% (*p* = 0.972)	*p* = 0.381	6	1.19 (0.93–1.54)	2	1.21 (0.89–1.65)	1	1.27 (0.49–3.33)	4	1.20 (0.92–1.56)
Trauma characteristics												
Being trapped (yes)	6	1.81 (1.47–2.24)	0.0% (*p* = 0.664)	*p* = 0.784	3	1.68 (1.21–2.34)	5	1.75 (1.41–2.18)	2	1.85 (1.41–2.44)	5	1.89 (1.50–2.37)
fear (yes)	4	2.97 (1.78–4.95)	92.9% (*p* < 0.001)	*p* = 0.659	4	2.97 (1.78–4.95)	3	3.55 (1.90–6.63)	–	–	4	2.97 (1.78–4.95)
Injure (yes)	14	2.06 (1.33–3.19)	90.0% (*p* < 0.001)	*p* = 0.185	9	1.79 (0.92–3.50)	6	1.45 (0.80–2.62)	5	1.89 (0.79–4.54)	10	2.08 (1.22–3.56)
Witness injury/death (yes)	7	1.62 (0.92–2.85)	90.5% (*p* < 0.001)	*p* = 0.383	4	1.64 (0.59–4.54)	4	1.19 (0.70–2.03)	2	0.98 (0.45–2.11)	7	1.62 (0.92–2.85)
Bereavement (yes)	23	2.49 (2.04–3.04)	70.8% (*p* < 0.001)	*p* = 0.465	17	2.56 (1.97–3.32)	15	2.36 (1.89–2.93)	8	2.65 (1.78–3.93)	17	2.61 (2.07–3.29)
Post-trauma characteristics												
Social support (yes)	14	0.81 (0.74–0.89)	94.7% (*p* < 0.001)	*p* = 0.103	8	0.82 (0.74–0.91)	8	0.72 (0.65–0.80)	4	1.11 (0.83–1.48)	8	0.83 (0.76–0.92)
Employment (no)	14	2.07 (1.49–2.88)	85.5% (*p* < 0.001)	*p* = 0.996	12	2.19 (1.50–3.20)	10	2.34 (1.59–3.43)	6	2.61 (1.55–4.39)	12	2.31 (1.63–3.29)
Loss of property (yes)	11	1.67 (1.31–2.15)	60.2% (*p* = 0.005)	*p* = 0.005 *	9	1.68 (1.25–2.25)	7	1.40 (1.15–1.71)	4	1.47 (1.07–2.02)	10	1.58 (1.24–2.03)
House damage (yes)	21	1.87 (1.52–2.30)	60.6% (*p* < 0.001)	*p* = 0.475	17	2.12 (1.71–2.63)	13	1.92 (1.49–2.49)	8	2.06 (1.45–2.94)	16	1.98 (1.58–2.48)
Involved in rescue (yes)	2	2.30 (0.39–13.57)	98.1% (*p* < 0.001)	–	2	2.30 (0.39–13.57)	2	2.30 (0.39–13.57)	1	5.69 (4.04–8.00)	1	5.69 (4.04–8.00)

***** trim and fill: Age (older) 1.05 (0.96–1.15); Loss of property (yes) 1.30 (1.01–1.68).

**Table A3:** Risk Factors for PTSD after earthquakes in children.

Factors	All Studies				High Quality		Adjustment		Within 6 Months		High Magnitude	
	*n*	OR (95% CI)	*I*^2^ (*p* Value)	Egger Test	*n*	OR (95% CI)	*n*	OR (95% CI)	*n*	OR (95% CI)	*n*	OR (95% CI)
Basic characteristics												
Age (older)	7	1.34 (1.12–1.61)	42.2% (*p* = 0.109)	*p* = 0.402	6	1.40 (1.15–1.71)	5	1.43 (1.16–1.76)	4	1.38 (1.06–1.78)	6	1.40 (1.15–1.70)
Gender (female)	20	1.45 (1.31–1.60)	26.5% (*p* = 0.135)	*p* = 0.138	16	1.47 (1.33–1.62)	12	1.43 (1.20–1.70)	8	1.48 (1.21–1.81)	19	1.43 (1.29–1.60)
Education (higher)	13	1.57 (1.11–2.21)	77.7% (*p* < 0.001)	*p* = 0.115	12	1.55 (1.08–2.23)	7	1.36 (0.78–2.37)	5	1.38 (0.70–2.74)	13	1.57 (1.11–2.21)
Religion (yes)	1	0.98 (0.44–2.17)	-	-	1	0.98 (0.44–2.17)	1	0.98 (0.44–2.17)	1	0.98 (0.44–2.17)	1	0.98 (0.44–2.17)
Ethnicity (minority)	8	1.39 (0.97–1.98)	0.0% (*p* = 0.900)	*p* = 0.645	6	1.35 (0.94–1.94)	8	1.39 (0.97–1.98)	2	1.04 (0.36–2.99)	8	1.39 (0.97–1.98)
Prior trauma (yes)	1	0.94 (0.62–1.42)	-	-	1	0.94 (0.62–1.42)	1	0.94 (0.62–1.42)	1	0.94 (0.62–1.42)	1	0.94 (0.62–1.42)
Trauma characteristics												
Being trapped (yes)	9	1.94 (1.52–2.47)	0% (*p* = 0.812)	*p* = 0.463	8	1.97 (1.52–2.56)	7	2.21 (1.67–2.92)	4	1.90 (1.33–2.72)	9	1.94 (1.52–2.47)
fear (yes)	2	2.24 (1.52–3.32)	0.0% (*p* = 0.777)	–	2	2.24 (1.52–3.32)	1	2.32 (1.47–3.67)	1	2.04 (0.95–4.36)	2	2.24 (1.52–3.32)
injury (yes)	11	2.05 (1.67–2.52)	4.9% (*p* = 0.397)	*p* = 0.379	8	2.04 (1.59–2.62)	9	2.17 (1.69–2.80)	5	2.43 (1.77–3.35)	11	2.05 (1.67–2.52)
Witness injury/death (yes)	6	2.01 (1.44–2.80)	64.9% (*p* = 0.014)	*p* = 0.027 *	4	2.26 (1.59–3.22)	4	2.00 (1.32–3.04)	2	2.68 (1.59–4.54)	6	2.01 (1.44–2.80)
Bereavement (yes)	18	2.24 (1.95–2.56)	19.7% (*p* = 0.219)	*p* = 0.217	15	2.33 (2.02–2.69)	11	2.33 (1.82–2.99)	8	2.30 (1.72–3.07)	17	2.28 (1.97–2.65)
Post-trauma characteristics												
Social support (yes)	2	0.94 (0.85–1.03)	95.4% (*p* < 0.001)	-	-	-	2	0.94 (0.85–1.03)	1	0.89 (0.86–0.93)	2	0.94 (0.85–1.03)
Loss of property (yes)	11	1.76 (1.53–2.02)	0.0% (*p* = 0.696)	*p* = 0.276	10	1.93 (1.60–2.33)	7	1.63 (1.39–1.92)	4	1.80 (1.35–2.39)	11	1.76 (1.53–2.02)
House damage (yes)	9	1.38 (0.98–1.94)	55.1% (*p* = 0.023)	*p* = 0.308	7	1.32 (0.87–1.98)	8	1.22 (0.94–1.59)	6	1.17 (0.85–1.62)	9	1.38 (0.98–1.94)

***** Trim and fill: Witness injury/death (yes) 1.54 (1.12–2.13).
